# Translation, cultural adaptation and validation of the Danish version of the haematological malignancy patient-reported outcome measure (HM-PRO)

**DOI:** 10.1186/s41687-025-00869-2

**Published:** 2025-04-29

**Authors:** Sam Salek, Sören Möller, Niels Abildgaard, Tine Rosenberg, Maria Torp Larsen, Peter Asdahl, Kasper Kofod Pedersen, Marie Therese Lassen, Christen Lykkegaard Andersen, Lene Kongsgaard Nielsen

**Affiliations:** 1 School of Life and Medical Sciences, University of Hertfordshire, College Lane, Hatfield, SL9 8BL UK; 2 Open Patient Data Explorative Network (OPEN), Odense University Hospital, Odense, Denmark; 3 Department of Clinical Research, University of Southern Denmark, Odense, Denmark; 4 Quality of Life Research Center, Department of Haematology, Odense University Hospital, Odense, Denmark; 5 Department of Haematology, Copenhagen University Hospital, Rigshospitalet, Denmark; 6 Department of Haematology, Roskilde University Hospital, Roskilde, Denmark; 7 Department of Haematology, Aarhus University Hospital, Aarhus, Denmark; 8 Department of Haematology, Aalborg University Hospital, Aalborg, Denmark; 9 Research Unit for General Practice, Department of Public Health, University of Copenhagen, Copenhagen, Denmark; 10 Section of Haematology, Department of Internal Medicine, Gødstrup Hospital, Herning, Denmark

**Keywords:** Patient-reported outcomes, Danish HM-PRO, Malignant lymphomas, Quality of life, Cross-cultural adaptation

## Abstract

**Background:**

Assessment of cancer patients´ quality of life (QoL) through patient-reported outcomes (PRO) during and after treatment is gaining ground. The HM-PRO is the first generic Haematological Malignancy specific PRO measure for use in clinical practice and clinical trials. Such generic tools are needed in Denmark. The study aim was to translate and cross-culturally adapt the HM-PRO into Danish and evaluate the psychometric properties.

**Methods:**

Translation and cross-cultural adaptation of the original English HM-PRO into Danish followed established guidelines. After cognitive debriefing interviews, it underwent psychometric testing with a variety of hematologic malignancies. Construct validity, internal consistency, dimensionality, item response theory (IRT) and differential item functioning were investigated.

**Results:**

295 patients were included for psychometric evaluation; confirmatory factor and bifactor analyses for both HM-PRO parts provided good evidence to support the suggested factor structure (Cronbach’s-α Part-A = 0.81, Part-B = 0.84; Part-A CFA CFI = 0.922, TLI = 0.912; bi-factor CFI = 0.989, TLI = 0.978). IRT showed good item-fit and factor loadings and absence of local dependency.

**Conclusion:**

The HM-PRO has demonstrated favourable psychometric properties and can be used broadly within the Danish Healthcare system to monitor symptoms as well as QoL impact of patients with haematological cancer and optimize patient engagement during routine cancer care.

**What is the new aspect of your work?:**

In response to the intention of the Danish Health Authority to systematically collect PRO data on health-related QoL in Danish cancer patients, this study investigates the translation and cross-cultural adaptation of the original English HM-PRO into Danish.

**What is the central finding of your work?:**

Few issues were met with the translation and adaptation of HM-PRO into Danish.

**What is (or could be) the specific clinical relevance of your work?:**

The HM-PRO has demonstrated favourable psychometric properties and can be used broadly within the Danish Healthcare system to monitor symptoms as well as QoL impact of patients with haematological cancer and optimize patient engagement during routine cancer care.

**Supplementary Information:**

The online version contains supplementary material available at 10.1186/s41687-025-00869-2.

## Introduction

Assessment of cancer patients’ quality of life (QoL) through patient-reported outcomes (PRO) during and after treatment is gaining ground. In fact, implementation of PROs into clinical practice is a strategic target of leading cancer organizations both in Europe and in the United States [1]. Patients with haematological malignancies will often be highly affected by disease symptoms and/or side effects to treatment. To capture this, systematic use of PROs may help to better understand the actual symptom and treatment burden experienced by patients themselves. Further, the use of PROs in routine clinical practice may be highly beneficial for improving patient-physician communication and to prioritize patient-centred care [2, 3], thereby improving the QoL in patients affected by haematological malignancies. In Europe, one of the most widely used cancer PRO instrument is the EORTC QLQ-C30 questionnaire, which is regarded as a cancer generic questionnaire for use in solid cancer clinical trials [4]. Also, the EORTC QLQ-C30 has been used as a treatment effect measure in several haematological trials [5]. However, recent studies in patients with haematological malignancies have shown that some symptoms are unique to this subgroup of cancer patients, and may thus not be captured by the existing generic cancer questionnaires, originally designed for use in solid cancers [6, 7]. Therefore, the “Scientific Working Group for Quality of Life and Symptoms”, within the European Haematology Association, decided to develop a new generic PRO designed specifically for and by patients with haematological malignancies (the HM-PRO) [8, 9]. With increasing importance of measuring QoL in patients with haematological malignancies including issues that are important to patients, a new validated questionnaire could be an important instrument for both clinicians and patients in daily clinical practice as well as in clinical trials.

The HM-PRO consists of 42-items, divided into two parts (A and B). Part A comprises 24 items covering daily impacts on patients´ QoL. It is grouped into four subdomains; Physical well-being (7 items), Social well-being (3 items), Emotional behaviour (11 items), and Eating and Drinking (3 items). The recall period for part A is “today”. Part B consists of 18 items and constitutes a single domain targeting patient-experienced disease symptoms and treatment side effects specifically related to haematological malignancies. The recall period for part B is the “past 3 days”. Items in Part A and B are rated on a 3-point Likert scale ranging from “not at all” (0 point) to “a lot” (2 points). In addition, a “not applicable” (0 point) response option is available too in Part A and is incorporated in the calculation of the subdomain scores in Part A. Subdomain scores are standardized to a 0 to 100-point scale, with higher scores meaning greater impact on QoL and a higher symptom burden. No total HM-PRO score is calculated [10].

Within the past two decades, the Danish Health Authority has launched five national cancer plans with the aim of improving cancer treatment across all diagnoses. In the fourth cancer plan (Cancer Treatment Package IV) from 2016 (www.sst.dk), it is specified that the use of PROs in clinical care should be optimized. The intention is to systematically collect PRO data on health-related QoL in Danish cancer patients, including those affected by haematological malignancies. It is an expressed goal to further incorporate PRO data into the National Danish Clinical Registries, and therefore, a validated robust and adequate generic PRO for patients with haematological malignancies is highly needed.

Advances in the treatment of haematological cancers have led to improvement in survival putting emphasis on the long-term effects of the disease and its treatments affecting the health‐related quality of life (HRQoL) of HM patients both short term and long term. Difficulty with physical and psychosocial activity, living with uncertainty, worrying about future and relapse and impact on work life are evident in survivors of HM and bone marrow transplant.

A comprehensive list of quality-of‐life issues important to patients suffering from haematological malignancies was presented in a systemic review and a list of HRQoL instruments used in haematological malignancies in both daily clinical practice and research provided [6]. However, none of the instruments reviewed (except MyPOS) appeared to have been developed specifically for patients with HM and for use in clinical practice. Evidence suggests that the psyche of patients with HMs is different to that of patients with solid tumours, and the conceptual model for the two is quite different. MH patients experience significant challenges of emotional/psychological disturbances, prolonged periods of neutropenia, more frequent visits to intensive care units, are more likely to die in hospital, receive less information on the sexual side effects of treatment and are much less likely to be referred to specialist palliative care at the end of their illness [6].

The aim of this study was (1) to translate and cross-culturally adapt the HM-PRO into Danish and (2) to psychometrically evaluate its performance in Danish patients with haematological malignancies.

## Materials and methods

### Study design

This study was a nationwide project with participation from six haematology departments across Denmark sponsored by the Department of Haematology, Odense University Hospital, Denmark. The study included three steps; (1) translation and cross-cultural adaptation, (2) cognitive debriefing and (3) psychometric testing. According to Danish law, this study was exempted for notification to the regulatory authorities. The study was approved by the Danish Data Protection Agency (Journal number 19/13870).

### Translation, cross-cultural adaptation and cognitive debriefing

Translation and cross-cultural adaptation of the original English HM-PRO into Danish was conducted and followed standardised guidelines for forward and backward translation procedures [11–14] (detailed methodology is given in the supplementary materials). Cognitive debriefing interviews were conducted on 11 patients.

### Psychometric testing of the Danish version of the HM-PRO

Subsequently, the approved Danish version of the HM-PRO was empirically tested for structural validity to confirm a number of constructs of the original English HM-PRO. For this, participants were recruited consecutively at the aforementioned six departments and asked to complete the Danish HM-PRO once. Both inpatients and outpatients were eligible for participation. Besides being diagnosed with one of the haematological malignancies as listed below the inclusion criteria for participation were: 18 years or older, able to understand and read Danish, and sufficient cognitive function (subjectively decided by a responsible healthcare professional). Participants were excluded if they had known comorbidities resulting in a high level of symptoms not related to their haematological disease and/or if they had an expected remaining life of less than 3 months from study enrolment.

To achieve a wide range of the most common haematological diagnoses in accordance with the generic characteristics and constructs of the HM-PRO, eligible participants followed the 2008 WHO classification of tumours of haematopoietic and lymphoid tissues [15], being:

- Acute myeloid leukaemia and related precursor neoplasms including therapy-related myeloid neoplasms and myeloid sarcoma (AML)
- Acute lymphoid leukaemia) (ALL)
- Chronic lymphocytic leukaemia (CLL)
- Chronic myeloid leukaemia (CML)
- Multiple myeloma (MM)
- Aggresive non-Hodgkin’s lymphoma (ANHL)
- Indolent non-Hodgkin’s lymphoma (INHL)
- Hodgkin’s lymphoma (HL)
- Myeloproliferative neoplasm (MPN)
- Myelodysplastic (MDS)

### Data collection procedure

At each of the six participating departments, a site investigator was responsible for the recruitment of participants. Eligible participants were provided with oral and written study information. Patients who accepted to participate signed an electronic consent form prior to data collection. To ensure an equal distribution of participants in accordance with the ten haematological sub diagnoses, the overall participant characteristics were monitored as the process of inclusion progressed and gave input to the site investigators about the current recruitment status in terms of diagnoses and sociodemographic variation. As such, the site investigators were able to target their recruitment of participants in accordance with the recruitment strategy.

Sociodemographic characteristics and clinical data of the participants were collected via self-report as well as physician-report. Self-reported data using a tablet concerned the following; age, gender, education (highest grade completed), and known comorbidity. Physician-reported data, extracted from medical records, concerned the following; cancer type, time since diagnosis, treatment history, current treatment, and ECOG performance status.

### Data management

Data were stored in REDCap, an electronic web-based data capture system, hosted by Open Patient data Exploratory Network (OPEN) at Odense University Hospital, Denmark. The REDCap system managing the electronic data collection was designed so that participants were required to answer all questions, thus resulting in no missing data. Patients who were unwilling/unable to complete the HM-PRO electronically were allowed to self-complete the survey using hard copy.

### Statistical analyses

Descriptive statistics included mean and standard deviation (SD). Correlation and regression coefficient for each item were presented. All statistical analyses were performed using STATA 15.0 software, with probability of Type I error set at *p* < 0.05 level.

### Validity

Validity was measured by construct validity, which is defined as the degree to which the scores of a measurement instrument are an adequate reflection of the dimensionality of the construct to be measured. As exploratory analysis of the factor structure and number of dimensions/scales was performed during the development of the original HM-PRO [9], confirmatory factor analysis (CFA) was used in this study to examine whether the data fit the predetermined model [16]. No listwise or pairwise deletion was carried out. Instead, a likelihood ratio method, e.g. full information maximum likelihood (FIML) or expectation-maximization algorithm (EM) was used to calculate estimates on all available data.

According to COSMIN, the minimum requirement of a sample size to conduct CFA is 7 subjects per item [17, 18]. As the HM-PRO is a 42-item questionnaire, a sample size of 294 participants was thus the target for this CFA. To comply with the intention of an equal distribution of haematological malignancies, attempts to recruit 42 patients from each of the seven haematological subgroups (as described above) were made. This applied also to variation in socio-demographic variables (e.g. *age*,* gender*,* educational levels*) and disease states (i.e. stable, remission, progression).

The HM-PRO data were not normally distributed, therefore for CFA and bi-factor modelling the Weighted Least Squares with Mean and Variance adjusted (WLSMV) estimator [19, 20] was used that is designed for ordinal data, is asymptotically distribution free, and does not make distributional assumptions about the observed variables. Fit parameters scaled for the WLSMV were used to test whether the proposed model was superior to alternative models. CFA and IRT were used to test proposed models and evaluation of model fit was performed using the following fit parameters. Root Mean Square Error of Approximation (RMSEA) expressing the lack of fit per degree of freedom of the model with values interpreted as follows: ≤0.05 = very good; >0.05–0.099 = good; ≥0.10 poor fit. The comparative fit index (CFI) assesses fit relative to a null model and ranges from 0 to 1 with values of 0.90–0.95 indicating acceptable and > 0.95 good fit. Tucker–Lewis index (TLI) adjusts for the number of model parameters and is interpreted as for CFI. The Standardised Root Mean Square Residual (SRMR) including 90% confidence intervals (CI) is the average of the differences between the observed and predicted correlations and has a range from 0 to 1 with values < 0.08 indicating good fit. Internal consistency was determined by Cronbach’s alpha [21, 22] for Part A (and its domains) calculated using IBM SPSS Statistics version 27.

Bi-factor analysis was performed using R version 4.2.2 (R Foundation for Statistical Computing) with package Lavaan version 0.6–16. The estimator used was maximum likelihood (ML) and missing values were handled with full information maximum likelihood (FIML) estimation for latent variables. The Omega function in Lavaan was used to guide selection of factors for bi-factor modelling, additionally using factor loading.

Differential Item Functioning (DIF) analyses were performed across two variables: gender and age. An age cut-off at 69 years (i.e. median) was used to provide balanced groups, and male vs. female was used as per the data. Item response theory for DIF was performed by IRTPRO version 4.2 (Scientific Software International Inc., Chapel Hill, NC, USA), using a Graded Response Model (GRM) with 3 categories. Independent models were run for Part A (24 items) and Part B (18 items).

## Results

### Translation and cross-cultural adaptation

The forward and backward translations did not provide any major issues. However, minor issues, mostly related to cross-cultural matters, were solved through consensus with participation from both forward and backward translators. e.g., the item “I feel distressed” had to be translated into Danish with the use of adjectives (Jeg er frustreret/ramt/ulykkelig), as the Danish language do not have a single word that covers all the constructs of being distressed. Thus, the Danish version of this item contained three adjectives separated with slashes, rather than commas, to ensure that patients did not have to feel affected by all three aspects as at the same time to be able to correctly rate their level of distress.

### Cognitive debriefing

The cognitive debriefing interviews were conducted on 11 patients in total (4 females; 3 newly diagnosed, 7 stable/in remission and 1 in relapse; 4 aged < 50, 6 aged between 50 and 80 and 1 aged > 80; and 3, 2 and 6 with low, mid and high educational status, respectively. The diagnoses were as follows: HL = 2, MDS = 2, MPN = 1, AML = 3, MM = 1, and DLBCL = 1.

Only item number 7 in the emotional domain (*I do not feel confident*) needed careful consideration and discussion. Three of eleven patients mentioned this item to be confusing and difficult to answer. This was due to the item being formulated differently compared to all the other items. For example, it is phrased in a reversed manner to comply with the fact that all the items of the HM-PRO concerns impairment and, as such, this item uses negative connation. This created doubts on how to answer the question correctly. Thus, to emphasize that this item needs special attention before answering (e.g. due the negative wording) a note in brackets was added to this item in the Danish version and now reads “Jeg føler mig ikke selvsikker (vær opmærksom på, at der står IKKE)”. This revised Danish version of this item was tested on four patients and no further concerns were raised. A final version of the Danish HM-PRO was approved in August 2019.

### Psychometric validation

#### Patient characteristics

From August 2019 to June 2020, 295 participants signed up for inclusion for the psychometric validation of the Danish HM-PRO. Recruitment of patients from the six departments was distributed as follows: Aalborg 70, Aarhus 49, Odense 39, Roskilde 28, Herlev 71, and Copenhagen University Hospital 44 patients. However, despite signed consent, the HM-PRO was left empty in one case, and in another case the HM-PRO was only partly completed. The sample for complete-case analyses thus constituted 293 patients. The majority was outpatient, constituting 75% of the total sample (222/293). Mean age was 65 years (SD 15) and 137 (47%) were females (Table 1). The largest hematologic group was patients with aggressive non-Hodgkin’s lymphoma constituting 17% of the total sample (51/293) (Table 1). A certain number of study participants requiring assistance with completing any PRO instrument is not unusual. With reference to this study in particular, there were some people with limited visual ability who needed assistance to complete questionnaires or who needed additional explanation for using an electronic device (Table 1), rather than on the questionnaire itself, but this did not affect validity testing of the Danish HM-PRO in any way.

**Table 1:** Demographic data and clinical characteristics (*n* = 293)

	*n* (%)
**Age**, mean (SD)	64.8 (15)
Median	69
Range	19–92
**Sex**	
-Female	137 (47)
-Male	156 (53)
**Setting (at questionnaire completion)**	
-Inpatient	71 (25)
-Outpatient	222 (75)
**Educational level**	
-Elementary school	47 (16)
-Vocational basic course	110 (38)
-Medium cycle higher education (3–4 years)	87 (30)
-Long cycle higher education (> 4 years)	49 (17)
**Civil status**	
**-**Unemployed (looking for job)	5 (2)
-Employed (full-time)	47 (16)
-Employed (part-time)	15 (5)
-Student	6 (2)
-Retired	187 (64)
-On sick leave	33 (11)
**Haematological diagnosis**	
- Acute myeloid leukaemia	27 (9)
- Acute lymphoid leukaemia	14 (5)
- Chronic lymphocytic leukaemia	32 (11)
- Chronic myeloid leukaemia	17 (6)
- Multiple myeloma	44 (15)
- Aggresive non-Hodgkin’s lymphoma	51 (17)
- Indolent non-Hodgkin’s lymphoma	31 (11)
- Hodgkin’s lymphomah	15 (5)
- Myeloproliferative neoplasm	34 (12)
- Myelodysplastic syndrome	26 (9)
**ECOG (for the outpatient patients only)**	
-0	98 (44)
-1	96 (43)
-2	19 (9)
-3	6 (3)
-4	1 (< 1)
Missing	1 (< 1)
**Time since diagnose (months)**,** mean (SD)**	
≤ 6 months	78 (27)
6–12 months	32 (11)
12–36 months	58 (20)
> 36 months	120 (41)
Missing	5 (2)
**Disease status**	
-Stable	118 (40)
-Remission	74 (25)
-Progression	93 (32)
Missing	8 (3)
**In active treatment (at questionnaire completion)**	
-yes	205 (71)
-no	84 (29)
If yes, which treatment:	
- Allogenic transplantation	2 (1)
- Autologous transplantation	3 (2)
- Chemotherapy	149 (73)
- Non-chemotherapy	41 (20)
- Other	10 (4)
**HM-PRO completion**	
- Hard copy	32 (11)
- Electronic	261 (89)
- Needed assistance with completion of the electronic version	64 (22)
- Needed assistance with reading out loud the questions	37 (13)

### Validity

#### Internal consistency

Internal consistency as calculated by Cronbach’s alpha is shown in Table 2.

**Table 2:** Internal consistency of the HM-PRO

HM-PRO	HM-PRO domains	This study (Danish)	Goswami ^1^	Revised version ^2^	Assessment 1 (t0) ^2^	Assessment 2 (t7) ^2^
**Part A**	Overall Part A (24 items)	0.81	0.95	0.91	0.90	0.90
	PB = Physical behaviour (7 items)	0.66			0.88	0.91
	SW = Social wellbeing (3 items)	0.46			0.70	0.68
	ES = Emotional state (11 items)	0.72			0.85	0.87
	ED = Eating/Drinking (3 items)	0.78			0.84	0.85
**Part B**	All items (18 items)	0.84	0.85	0.76	0.80	0.80

^1^Final revised version: Goswami et al. [9]. ^2^ Development versions: Goswani [23]

#### Structural validity

There was no missing data in the dataset used for psychometric analysis. The CFA of the original 4-factor model for Part A of the HM-PRO revealed a RMSEA of 0.077 (90%CI 0.070–0.084), a SRMR value of 0.095, and a CFI and TLI of 0.92 and 0.91, respectively (Table 3). This model was superior to two explorative 3-factor models where the physical and social domains, and the social and emotional domains, respectively were combined into one domain (Table 3). The CFA of the 1-factor model for Part B of the HM-PRO revealed a RMSEA of 0.06 (90%CI 0.05–0.07 ), and a CFI and TLI of 0.94 and 0.93, respectively (Table 3).

**Table 3:** The model statistics of part A and part B of the Danish version of the HM-PRO

Model Part A	X^2^	*P*	CFI	TLI	RMSEA (90% CI)	SRMR
4 factor model (the original model) - Complete case analysis	623.45	< 0.001	0.922	0.912	0.077 (0.070–0.084)	0.095
3 factor model - Physical and Social domains combined	726.58	< 0.001	0.910	0.901	0.082 (0.075–0.088)	0.105
3 factor model - Social and emotional domains combined	695.14	< 0.001	0.913	0.903	0.080 (-0.74-0.087)	0.100

**Model Part B**	**X** ^**2**^	***P***	**CFI**	**TLI**	**RMSEA (90% CI)**	**SRMR**
1 factor model (the original model) - Including all patients that have answered on just some of the questionnaire	241.99	< 0.001	0.936	0.928	0.060 (0.050–0.070)	0.095

#### Bi-factor analyses

The bi-factor of the original 4-factor model for Part A revealed a RMSEA of 0.078 (90%CI 0.070–0.084), a SRMR value of 0.095, and a CFI and TLI of 0.92 and 0. 91, respectively (Table 4). The fit of this model was marginally better than the 4-factor CFA model. Bi-factor models based on factors taken from hierarchical models (Lavaan Omega, see supplementary Figs. 1–3) are also shown in Table 4. The 4-factor model based on the hierarchal model gave better fit than the original 4-factor model. It was not appropriate to fit a bi-factor model to Part B as it has been shown to be unidimensional.

**Table 4:** The model statistics of part A bifactor of the Danish version of the HM-PRO

Model	X^2^	*P*	CFI	TLI	RMSEA (95% CI)	SRMR
4 factor bifactor model (the original model)^1^	623.45	< 0.001	0.922	0.912	0.077 (0.070–0.084)	0.095
2 factor bifactor model from Omega^2^	304.81	< 0.001	0.962	0.953	0.056 (0.048–0.064)	0.071
3 factor bifactor model from Omega^3^	265.69	< 0.001	0.971	0.965	0.048 (0.040–0.057)	0.066
4 factor bifactor model from Omega^4^	182.77	< 0.001	0.983	0.978	0.038 (0.028–0.047)	0.054

PB = Physical, behaviour, SW = Social wellbeing, ES = Emotional state, ED = Eating/drinking

^1^factor1 = PB1+PB2+PB3+PB4+PB5+PB6+PB7, factor2 = SW1+SW2+SW3, factor3 = EB1+EB2+EB3+EB4+EB5+EB6+;EB7+EB8+EB9+EB10+EB11, factor4 = ED1+ED2+ED3

^2^factor1 = PB1 +PB2+PB3+PB4+PB5+PB6+PB7+ED1+ED2+ED3+SW1+EB1+EB9+EB10, factor2 = EB1+EB2+EB3+EB4+EB5+EB6+EB7+EB8+EB9+EB10+EB11+SW1+SW2+PB6

^3^factor1 = EB1+EB2+EB3+EB4+EB5+EB6+EB7+EB8+EB9+EB10+EB11+SW2+SW3, factor2 = PB1+PB2+PB3+PB4+PB5+PB6+PB7+SW1, factor3 = ED1+ED2+ED3

^4^factor1 PB6+EB1+EB2+EB3+EB7+EB10+SW1+SW2+SW3, factor2 = PB1+PB2+PB3+PB4+PB5+PB7, factor3 = EB4+EB5+EB6+EB8+EB11, factor4 = ED1+ED2+ED3+EB9

#### DIF analyses

Item response theory with a dataset *N* = 293 was used to investigate DIF. All factor loadings > 0.36 and almost all local dependency (LD) statistic < 10 indicated minimal local dependency [24]. For Part A, S-X^2^ item level diagnostic statistics indicated optimal fit for items with *p* > 0.05 for all items and a RMSEA of 0.07. Item trace lines indicated misfitting of items PB6, PB7, and SW3, attributable to missing data. For Part B, S-X^2^ item level diagnostic statistics indicated a suboptimal fit for items 3 (SS3) only, with *p* < 0.05 and a RMSEA of 0.04. Item trace lines indicated misfitting of item SW 4 only.

The DIF by gender on Part A was performed with Group 1 (male) = 155 and Group 2 (female) = 137. All factor loadings in both groups > 0.42. From S-X^2^ item level diagnostic statistics only item 12 (EB2) in both groups had suboptimal fit with *p* < 0.05. The DIF analysis by X^2^ testing showed overall DIF in items 10 (SW3), 13 (EB3) and 16 (EB6) due location (uniform) DIF (*p* < 0.05). RMSEA was 0.0 (Table 5).

DIF by age for Part A with Group 1 = 145 (age < 69) Group2 = 147 (age > = 69) showed uniform DIF in Items 1 (PB1), 2 (PB2), 5 (PB5), 12 (EB2) and 16 (EB6), discrimination/non-uniform DIF in items 10 (SW3), 13 (EB3), 20 (EB10), and 23 (ED2), and both uniform and non-uniform DIF in items 6 (PB6) and 22 (ED1) (Table 5).

**Table 5:** DIF statistics for graded items part A by gender and age

DIF statistics for graded items Part A by gender											
Item	Group 1	Group 2	Total X^2^	d.f.	*p*	X^2a^	d.f.	*p*	X^2c/a^	d.f.	*p*
SW3	10	10	24.6	3	0.0001	1.7	1	0.1877	22.8	2	0.0001
EB3	13	13	18.5	3	0.0003	1.4	1	0.2353	17.1	2	0.0002
EB6	16	16	11.5	3	0.0091	1.4	1	0.2352	10.1	2	0.0063

**DIF statistics for graded items Part A by age**											
**Item**	**Group 1**	**Group 2**	**Total X** ^**2**^	**d.f.**	**p**	**X** ^**2a**^	**d.f.**	**p**	**X** ^**2c/a**^	**d.f.**	**p**
SS1	1	1	8.2	3	0.0422	0.0	1	0.9508	8.2	2	0.0167
SS3	3	3	10.0	3	0.0186	0.0	1	0.8883	10.0	2	0.0068
SS8	8	8	9.9	3	0.0194	3.2	1	0.0723	6.7	2	0.0356

Total χ2 is overall DIF, χ2a for slope (discrimination, non-uniform), χ2c/a for location (uniform)

DIF by gender on Part B was performed with the same grouping and showed no DIF in any items. RMSEA was 0.03. DIF by age for Part B with the same grouping showed uniform DIF for items 1 (SS1), 3 (SS3), and 8 (SS8). RMSEA was 0.04 (Table 6).

**Table 6:** DIF statistics for graded items part B by age

Item	Group 1	Group 2	Total X ^2^	d.f.	*p*	X ^2a^	d.f.	*p*	X^2c/a^	d.f.	*p*
SS1	1	1	8.2	3	0.0422	0.0	1	0.9508	8.2	2	0.0167
SS3	3	3	10.0	3	0.0186	0.0	1	0.8883	10.0	2	0.0068
SS8	8	8	9.9	3	0.0194	3.2	1	0.0723	6.7	2	0.0356

## Discussion

In the present study, we have translated and cross-culturally adapted the HM-PRO into Danish and tested its psychometric properties in Danish patients with a variety of haematologic malignancies while retaining the same factor structure as the original version of the HM-PRO. Few issues were met with regards to the translation and adaptation of the HM-PRO into Danish. After cognitive debriefing, a revised version of this item was tested on four patients. No further concerns were raised and the final version of the Danish HM-PRO was approved.

### Confirmatory factor analysis

The CFA for both parts of the HM-PRO provided evidence to support the suggested factor structure of the HM-PRO. In the present study, the RMSEA was within the acceptable levels of error of approximation, while the SRMR value was slightly below the cut-off, thus not indicating a poor fit. With regards to the CFI and TLF values, these were slightly outside the suggested range, but within acceptable distance to support the model fit. This is based on the fact that the guidelines for acceptable model varies and as the sample size was at the lower end of the suggested numbers for CFA. Thus, future studies should aim to include even larger sample sizes.

Internal consistency as calculated by Cronbach’s alpha was 0.81 for Part A and 0.84 for Part B of the Danish HM-PRO confirming the internal consistency of both scales [25]. It should be noted that it is expected that the magnitude of Cronbach’s alpha is related to the number of items used in its calculation, and will therefore increase as the number of items in the calculation increases [17]. This effect often tends to make domains with few items such as the social/wellbeing and eating/drinking with three items each to “underperform” and those with many items “overperform”. However, these items came from patient consultation with 130 patients, and when the item list was saturated and duplicate items removed, those were clearly related conceptually combined into their own domains and validated, first by EFA then by CFA, so these domains with few items are a real construct in capturing QoL of haematological malignancies.

### BIfactor and DIF analysis

Bifactor analysis for Part A of the HM-PRO showed slightly better fit for the original 4-factor model than the CFA analysis, and a slightly modified 4-factor bifactor model based on a hierarchical model improved this further. Interestingly, the hierarchical model which is completely unspecified except for the number of factors to obtain, using only the patient responses with no predetermined relationships, mapped very closely to the original domains hypothesised during development [10], and confirmed by CFA.

Generally, items showed good fit in the IRT models with minimal local dependency. The DIF analysis showed limited differential item functioning by gender. This would be expected as this is a baseline study of patients already diagnosed with haematological malignancy (so incidence, prognosis, response to treatment and survival are not relevant). Sztankay et al. reviewed gender differences in QoL in patients with haematological malignancies [26] and found female gender proved to be one predictor of impaired overall QoL in lymphoma patients, while representing the only variable associated with lower global QoL in a sample with chronic lymphocytic leukaemia (CLL). Diminished functioning and poor adjustment in occupational and social domains are equally well documented in female patients with HL or CLL. Previous study has explored the impact of gender on distress, anxiety, and depression in haematological cancer patients receiving chemotherapy [27] and found significant interaction between gender and practical and physical problems. The DIF by gender might be attributable to differences in perception of pain [28] and the increased risk of depression in women [29]. Both of these factors are significant predictors of QoL in female cancer patients [30].

However, when DIF analysis was performed on groups by age, significant DIF, both non-uniform and uniform, was revealed. A study has previously shown that age has a significant negative correlation with global QoL, physical functioning and role functioning (using EQ-5D and EORTC QLQ‐C30 instruments) as well as increasing age was also associated with lowest QoL [31]. Priscilla et al. [32] reported similar findings of reduced QoL with impaired physical and role functioning in older patients with a haematological cancer, and the trend reported by Slovacek et al. [33]

### Strengths

A strict translation guideline was followed including independent forward and backward translations [14]. This nationwide study with inclusion of patients with a variety of hematologic malignancies, sex, age, educational level, etc.) is a representative population for Danish patients with hematologic malignancies, thus improving the generalisability of the study. Furthermore, rigorous psychometric testing applied throughout the development of the Danish HM-PRO should provide the clinicians as well as the researchers with full confidence in the reliability and validity of the generated scores and its application to aid treatment decision-making.

### Limitations

The sample size for this study was smaller than desired for the analyses performed resulting in the fits for CFA (RMSEA, CLI and TLI) slightly below desired cutoffs. Also, although responsiveness values as well as other measures of validity, score bands systems and ‘Minimum important difference’ (MID) are available from studies of the source HM-PRO version, these were not assessed in the present study. As psychometric properties, in general, are context and population dependent, these values should also be investigated in a Danish context. Although equivalency was shown between electronic and paper versions during psychometric testing of the original HM-PRO [34], this should be further explored in the Danish version. Moving forward with the use of the Danish HM-PRO, there will be a need to test its responsiveness or longitudinal application with appropriately designed studies and these are being currently conducted within Denmark.

## Conclusions

The HM-PRO is the first generic haematological specific PRO measure for use in clinical practice and clinical trials. Such a generic tool for health-related QoL measurement is needed in Denmark for several reasons. Among others, The Danish Government and Danish Regions aim to use PROs broadly within the Danish healthcare system: First, to increase and improve symptom monitoring during and after cancer treatment; and second, to optimize patient engagement during routine cancer care. One rationale for this is that routine use of PRO data has been found to clinically benefit patients during cancer treatment [35, 36].

However, since health-related QoL of patients with haematological malignancies differ from patients with solid tumours, there is a need for specific and generic tools developed specifically for haematological patients [6]. Feasibility in PRO data collection in daily clinical practice and minimization of patient burden has to be considered before implementation of PRO data in provision of healthcare. Therefore, a PRO instrument that can be used to monitor symptoms during routine cancer care as well as quality assessment of treatment through The Danish Clinical Registries will be of utmost importance.

The HM-PRO has demonstrated favourable psychometric properties for use in clinical practice, symptoms monitoring in registries as well as in clinical trials. Although the HM-PRO is a relatively new instrument, it has already undergone cross-cultural translation and been adapted into at least 22 other languages (https://hmpro.co.uk).

## Electronic supplementary material

Below is the link to the electronic supplementary material.

Supplementary Material 1

## Data Availability

Data available on request due to privacy/ethical restrictions.
