# Supplementary material for: Translation, cultural adaptation and validation of the Danish version of the haematological malignancy patient-reported outcome measure (HM-PRO)
Source: J Patient Rep Outcomes. 2025 Apr 29;9:43. doi: 10.1186/s41687-025-00869-2 (PMC12040771; doi:10.1186/s41687-025-00869-2)
Supplement: Supplementary file 1 — Supplementary Material 1 [file 41687_2025_869_MOESM1_ESM.docx]

**Translation and cross-cultural adaptation of the HM-PRO**

The translation followed the procedure below:

1. Pre-preparation. Prior to the translation of the HM-PRO all translators were introduced to the content and meaning of the HM-PRO.
2. Forward translation. Two Danish natives fluent in English (bilingual expertise), independently translated the original English HM-PRO version into Danish.
3. Reconciliation. A third person (HRE), a native speaker of Dutch, reconciled the two forward translations. Discrepancies between the two forward translations were summarized into one document and the translators were then asked to choose the translation they found most suitable or come up with a revised translation. Subsequently, two persons (HRE and LKN), native speakers of Dutch, reconciled the summarized document including the forward translators agreements into ‘version one’ of the Danish HM-PRO.
4. Backward translation. Two persons with English (UK Nationality) as mother tongue and fluent in Danish independently translated the preliminary Danish HM-PRO back into English. The back-translators were blinded to the original English HM-PRO version.
5. Translation report. HRE forwarded a translation report, including the backward translated English version, to the corresponding author (SS) of the original English HM-PRO. SS reviewed and commented on the report and discrepancies between the original and the backward translated HM-PRO were solved by consensus.
6. Preliminary version of the Danish HM-PRO was prepared.
7. Cognitive debriefing. Using individual cognitive debriefing interviews the Danish HM-PRO was tested for readability, grammar and understanding, 11 with a haematological malignancy recruited at the university hospitals in Odense and Copenhagen, Denmark. To achieve a representative group of patients in terms of socio-demographic characteristics (gender, age and education), diagnosis and disease stage, patients were selected for inclusion ensuring that the group included 1) at least one patient from each of the ten haematological diagnostic groups, 2) patients being in active treatment and patients being off treatment, 3) minimum four patients who had experienced a first relapse/progression of their haematological malignancy, 4) at least five males and five females, 5) patients from each of the following age groups: < 50 years,50-80 years and >80 years, 6) patients with no formal education (basic or high school), patients with vocational education (e.g. mason, hairdresser, sales assistant), and patients with medium to higher education (e.g. school teacher, nurse, policemen, civil engineer, doctors).
8. Final report. The outcome of the translation and pilot-testing, including any reasons for refinement of the preliminary Danish HM-PRO version, was described in a short report and forwarded to SS for comparison to the original English version and for final approval of the Danish version of HM-PRO.

The semantic, experiential, conceptual and language differences between cultures are critical to be incorporated into adaptation of an instrument into another language and setting. These issues cannot be addressed in isolation, and thus the process of forward and backward translation, consolidation, and the specification of those conducting these steps ensures that these aspects and any other cultural and language differences are captured in the whole process. These processes, followed by cognitive debriefing is a confirmation that ensures the relevance of the new tool to the target population.

# **Copyrights of HM-PRO and publication policy**

Copyright of the Danish HM-PRO will remain with the original HM-PRO copyright holders. The final Danish HM-PRO can be used after permission granted by the copyright holders. The HM-PRO material will be treated in the strictest confidence as part of the protocol. The translation and validation protocol will be approved by Sam Salek before initiation of the project. The revised backward translation version of the HM-PRO will be approved by Sam Salek before starting cross-culture validation. Also, Sam Salek will approve the final Danish HM-PRO version based on the final report.

**Omega Heirarchical Analysis**

**Supplementary Figure 1. Part A 4 factor-hierarchical**

Alpha: 0.91 (Cronbach's alpha: main estimator of reliability)

G.6: 0.93

Omega Hierarchical: 0.65 (estimate the reliability of the general factor, controlling the variance of the specific factors i.e. true score variance in a composite that is attributable to the general factor)

Omega H asymptotic: 0.69

Omega Total 0.93 (joint reliability of all the factors of the model, without differentiating between the sources of variance of specific or general factors)

Explained Common Variance of the general factor = 0.47 (ratio of the general factor eigen value to the sum of all of the eigen values. As such, it is a better indicator of unidimensionality than of the amount of test variance accounted for by a general factor)

RMSEA index = 0.061 and the 10 % confidence intervals are 0.053 0.070, BIC = -667.78

**Supplementary Figure 2 Part A 3 factor- hierarchical**

Alpha: 0.91

G.6: 0.93

Omega Hierarchical: 0.61

Omega H asymptotic: 0.65

Omega Total 0.93

Explained Common Variance of the general factor = 0.48

RMSEA index = 0.073 and the 10 % confidence intervals are 0.065 0.080, BIC = -101.69

**Supplementary Figure 3 Part A 2 factor-hierarchical**

Alpha: 0.91

G.6: 0.93

Omega Hierarchical: 0.58

Omega H asymptotic: 0.63

Omega Total 0.92

Explained Common Variance of the general factor = 0.50

RMSEA index = 0.09 and the 10 % confidence intervals are 0.083 0.097, BIC = -527.35
